# Supplementary material for: SpatialProp: tissue perturbation modeling with spatially resolved single-cell transcriptomics
Source: bioRxiv. 2025 Dec 2:2025.11.30.691355. Preprint. [Version 1] doi: 10.64898/2025.11.30.691355 (PMC12822716; doi:10.64898/2025.11.30.691355)

APPENDIX

Table of Contents

Appendix	Description
A	Supplementary figures showing comprehensive benchmarking of the SpatialProp GNN module against the strong baseline of k-hop mean gene expression on the test graph and more complex GNN architectures.
B	List of genes and perturbation setup for mouse brain inflammatory signaling showcase of SpatialProp pipeline.
C	Table of interaction terms in CausalInteractionBench and associated Gene Ontology identifiers
D	Supplementary figures showing representative interaction terms with approximately zero or negative causal enrichment scores (CES)

Appendix A. Tables showing comprehensive benchmarking of the SpatialProp GNN module against the train global mean baseline, strong baseline of k-hop mean gene expression on the test graph, and more complex GNN architectures.

Spearman correlation between predicted and ground truth center cell gene expression

Dataset Identifier	SpatialProp (GNN)	Train Global Mean Baseline	Test 2-hop Mean Baseline	GNN (Nested with cell type prediction)	GNN (Cell type with global attention pool)	GNN (Cell type with predicted residual over 2-hop mean baseline)	GNN (Cell type with 3-hop graphs)
AC	0.684	0.586	0.660	0.682	0.680	0.631	0.680
AS	0.662	0.570	0.653	0.658	0.655	0.616	0.656
EX	0.681	0.589	0.666	0.678	0.678	0.632	0.676
RE	0.667	0.578	0.650	0.664	0.662	0.613	0.663
EA	0.282	0.276	0.286	0.274	0.265	0.210	0.267
MS	0.543	0.473	0.603	0.529	0.533	0.546	0.515
AD	0.212	0.240	0.203	0.227	0.213	0.093	0.220
AP	0.454	0.444	0.454	0.452	0.451	0.418	0.455
HD	0.634	0.476	0.590	0.631	0.631	0.574	0.631

Mean absolute error between predicted and ground truth center cell gene expression

Dataset Identifier	SpatialProp (GNN)	Train Global Mean Baseline	Test 2-hop Mean Baseline	GNN (Nested with cell type prediction)	GNN (Cell type with global attention pool)	GNN (Cell type with predicted residual over 2-hop mean baseline)	GNN (Cell type with 3-hop graphs)
AC	1.720	2.088	1.829	1.722	1.729	1.867	1.728
AS	1.855	2.244	1.945	1.863	1.872	2.017	1.878
EX	1.889	2.251	1.996	1.898	1.905	2.035	1.906
RE	1.848	2.234	1.956	1.857	1.862	2.018	1.861
EA	12.059	14.655	13.604	12.111	12.251	15.714	12.202
MS	2.446	2.772	2.272	2.495	2.485	2.407	2.533
AD	14.245	16.681	16.139	14.130	14.204	18.042	14.370
AP	2.650	2.936	2.846	2.660	2.649	3.039	2.610
HD	1.017	1.646	1.355	1.036	1.032	1.501	1.031

Appendix B. List of genes and perturbation setup for mouse brain inflammatory signaling showcase of SpatialProp pipeline.

Inflammation Response Genes (response calculated as sum of log1p expression of each gene in the list):

Stat1, Bst2, Jak1, Ifit1, Cdkn1a, Cdkn2a, C4b, H2-D1, H2-K1

Pro-inflammatory Perturbation Genes (perturbation modeled as ten-fold increased expression):

Ifng, Il6, Tnf, Il1a, Il1b, Jun, Apoe, B2m, C1qa, Cd69, Cd9, Lyz2

Appendix C. Table of interaction terms in CausalInteractionBench and associated Gene Ontology identifiers.

Interaction term	Production GO IDs	Response GO IDs
maltose	GO:0000024	GO:0034286, GO:0106081
l-histidine	GO:0000105	GO:0061460, GO:0090466, GO:1903810
l-tryptophan	GO:0000162, GO:0009096	GO:1904272
prostaglandin	GO:0001516, GO:0002539, GO:0031392, GO:0031393, GO:0031394, GO:0032306, GO:0032307, GO:0032308, GO:0032310, GO:0071810, GO:0071812	GO:0034694
histamine	GO:0001693, GO:0001694, GO:0001821, GO:0002349	GO:0034776, GO:0071420
cytokine	GO:0001816, GO:0001817, GO:0001818, GO:0001819, GO:0002367, GO:0002374, GO:0002375, GO:0002534, GO:0002718, GO:0002719, GO:0002720, GO:0002739, GO:0002740, GO:0002741, GO:0002742, GO:0002743, GO:0002744, GO:0042032, GO:0042035, GO:0042036,	GO:0034097

	GO:0042089, GO:0042107, GO:0042108, GO:0050663, GO:0050707, GO:0050710, GO:0050715, GO:1900015, GO:1900016, GO:1900017	
serotonin	GO:0001820, GO:0002351, GO:0014062, GO:0014063, GO:0014064, GO:0042427, GO:1905627, GO:1905628, GO:1905629	GO:1904014, GO:1904015
neurotransmitter	GO:0001956, GO:0007269, GO:0010554, GO:0046928, GO:0046929	GO:0099601
aldosterone	GO:0002018, GO:0032342, GO:0032347, GO:0032348, GO:0032349, GO:0035932, GO:2000858, GO:2000859, GO:2000860	GO:1904044, GO:1904045
retinoic acid	GO:0002138, GO:1900052, GO:1900053, GO:1900054	GO:0032526, GO:0071300
nitric oxide	GO:0002537, GO:0006809, GO:0045019, GO:0045428, GO:0045429	GO:0071731, GO:0071732
leukotriene	GO:0002540, GO:0019370, GO:0035490,	GO:0061737

	GO:0035491, GO:0035492	
peptide	GO:0002790, GO:0002791, GO:0002792, GO:0002793, GO:0043043	GO:1901652, GO:1901653
sucrose	GO:0005986	GO:0009744, GO:0106082
trehalose	GO:0005992	GO:0010353
inositol	GO:0006021, GO:1900088, GO:1900089, GO:1900090	GO:1902140, GO:1902141
chitin	GO:0006031, GO:0032883	GO:0010200, GO:0071323
sorbitol	GO:0006061	GO:0072708, GO:0072709
ethanol	GO:0006115	GO:0017036, GO:0045471, GO:0071361, GO:1901418
camp	GO:0006171	GO:0051591, GO:0071320
cgmp	GO:0006182	GO:0070305, GO:0071321
l-arginine	GO:0006526	GO:0010963, GO:0090467, GO:0097638, GO:1901042, GO:1902765, GO:1902826, GO:1902827, GO:1902828, GO:1903576, GO:1903577, GO:1905541, GO:1905542, GO:1905589
glycine	GO:0006545, GO:0061536	GO:0036233, GO:1900923, GO:1900924, GO:1900925, GO:1903804, GO:1905429, GO:1905430

proline	GO:0006561, GO:1902005, GO:1902006	GO:0010238, GO:0071235, GO:1902825, GO:1902834, GO:1902835, GO:1902836, GO:1905647
l-serine	GO:0006564	GO:1903812
octopamine	GO:0006589, GO:0061539	GO:0071927, GO:2000128, GO:2000129, GO:2000130
polyamine	GO:0006596, GO:0010967, GO:0170066	GO:0140202
fatty acid	GO:0000037, GO:0006633, GO:0042304, GO:0045717, GO:0045723	GO:0070542, GO:0071398
phosphatidylethanolamine	GO:0006646	GO:1905711, GO:1905712
cholesterol	GO:0006695, GO:0045540, GO:0045541, GO:0045542	GO:0070723, GO:0071397
ergosterol	GO:0006696, GO:0010895, GO:0032443, GO:0070452	GO:1901625
ecdysone	GO:0006697	GO:0035075, GO:0071390
bile acid	GO:0006699, GO:0032782, GO:0070857, GO:0070858, GO:0070859, GO:0120188, GO:0120189, GO:0120190	GO:0038183, GO:1903412, GO:1903413
progesterone	GO:0006701, GO:0042701, GO:2000182, GO:2000183, GO:2000184, GO:2000870,	GO:0032570

	GO:2000871, GO:2000872	
androgen	GO:0006702, GO:0035935, GO:2000180, GO:2000834, GO:2000835, GO:2000836	GO:2000825
estrogen	GO:0006703, GO:0035937, GO:1904076, GO:1904077, GO:1904078, GO:2000861, GO:2000862, GO:2000863	GO:0043627
glucocorticoid	GO:0006704, GO:0031946, GO:0031947, GO:0031948, GO:0035933, GO:2000849, GO:2000850, GO:2000851	GO:0051384
mineralocorticoid	GO:0006705, GO:0035931, GO:2000855, GO:2000856, GO:2000857	GO:0051385
farnesol	GO:0006715	GO:0097307, GO:0097308
glutathione	GO:0006750, GO:1903786, GO:1903787, GO:1903788	GO:0036347, GO:0072753, GO:0098709, GO:1901370
atp	GO:0006754, GO:0006758, GO:0006759, GO:1904669, GO:2001169, GO:2001170, GO:2001171	GO:0033198, GO:0071318
heme	GO:0006783, GO:0070453, GO:0070454,	GO:1904334

	GO:0070455, GO:0097037	
acetylcholine	GO:0008292, GO:0061526, GO:1905921, GO:1905922, GO:1905923	GO:1905144, GO:1905145
spermidine	GO:0008295, GO:1901304, GO:1901305, GO:1901307	GO:0140203
lipid	GO:0008610, GO:0046889, GO:0046890, GO:0051055, GO:0140353	GO:0033993, GO:0071396
pyridoxine	GO:0008615	GO:1903075
amino acid	GO:0008652, GO:0032973, GO:0044746, GO:0080143, GO:2000282, GO:2000283, GO:2000284	GO:0010237, GO:0010958, GO:0043092, GO:0043200, GO:0044745, GO:0089718, GO:1902837
methionine	GO:0009086	GO:0044690, GO:0061431, GO:1903692, GO:1904640
l-phenylalanine	GO:0009094, GO:0019274, GO:0019275	GO:0140925
l-leucine	GO:0009098, GO:2001276, GO:2001277, GO:2001278	GO:0043201, GO:0060356, GO:0071233, GO:1903128, GO:1903129, GO:1903130, GO:1903801, GO:1905532, GO:1905533, GO:1905534
l-valine	GO:0009099	GO:0090468, GO:1903805
glycoprotein	GO:0009101, GO:0010559,	GO:1904587, GO:1904588

	GO:0010560, GO:0010561	
biotin	GO:0009102	GO:0044756, GO:0070781, GO:0071296, GO:1901689, GO:1905135
lipopolysaccharide	GO:0009103	GO:0032496, GO:0071222
vitamin	GO:0009110	GO:0033273, GO:0071295
nucleoside	GO:0009163	GO:0180015
pyrimidine ribonucleotide	GO:0009220	GO:1905834, GO:1905835
thiamine	GO:0009228, GO:0070623, GO:0070624, GO:0090180	GO:0140125
menaquinone	GO:0009234	GO:0032572, GO:0071308
cobalamin	GO:0009236	GO:0033590, GO:0071297
peptidoglycan	GO:0009252, GO:0009285	GO:0032494, GO:0071224
amine	GO:0009309	GO:0014075
putrescine	GO:0009446	GO:1904585, GO:1904586
gamma-aminobutyric acid	GO:0009449, GO:0014051, GO:0014052, GO:0014053, GO:0014054	GO:0007214
gibberellin	GO:0009686, GO:0010371, GO:0010372, GO:0010373	GO:0009739
abscisic acid	GO:0009688, GO:0010115, GO:0010116, GO:0090359	GO:0009737
cytokinin	GO:0009691	GO:0009735
ethylene	GO:0009693, GO:0010364, GO:0010365, GO:0010366, GO:0042456	GO:0009723

jasmonic acid	GO:0009695, GO:0080141	GO:0009753
salicylic acid	GO:0009697, GO:0080142	GO:0009751
phenylpropanoid	GO:0009699	GO:0080184, GO:1905546
flavonoid	GO:0009813, GO:0009962, GO:0009963, GO:0009964	GO:1905395, GO:1905396
alkaloid	GO:0009821	GO:0043279, GO:0071312
auxin	GO:0009851, GO:0010600, GO:0010601	GO:0009733
amylopectin	GO:0010021	GO:0044591, GO:1900523
vitamin e	GO:0010189, GO:1904965, GO:1904966	GO:0033197, GO:0071306
brassinosteroid	GO:0010422, GO:0010423, GO:0016132, GO:2000488	GO:0009741
ketone	GO:0010566, GO:0042181	GO:1901654, GO:1901655, GO:1990369
vascular endothelial growth factor	GO:0010573, GO:0010574, GO:0010575, GO:1904046	GO:0038084, GO:1900746, GO:1900747, GO:1900748
norepinephrine	GO:0010700, GO:0010701, GO:0014061, GO:0042421, GO:0048243	GO:0071873
hydrogen peroxide	GO:0010728, GO:0010729, GO:0010730, GO:0050665	GO:0042542, GO:0070301
triglyceride	GO:0010866, GO:0010867, GO:0010868, GO:0019432	GO:0034014, GO:0071401
vitamin d	GO:0010957, GO:0042368,	GO:0033280, GO:0071305

	GO:0060556, GO:0060557	
dopamine	GO:0014046, GO:0014059, GO:0033602, GO:0033603, GO:0042416, GO:1903179, GO:1903180, GO:1903181	GO:1903350, GO:1903351
epinephrine	GO:0014060, GO:0032811, GO:0032812, GO:0042418, GO:0048242	GO:0071871
pantothenate	GO:0015940, GO:0033317, GO:0033318	GO:0044755, GO:0098717, GO:1901688
carbohydrate	GO:0006093, GO:0016051, GO:0033231, GO:0043255	GO:0009743, GO:0097319, GO:0098704
triterpenoid	GO:0016104	GO:1905836, GO:1905837
sterol	GO:0016126, GO:0106118, GO:0106119, GO:0106120	GO:0036314, GO:0036315
glycoside	GO:0016138, GO:0016141	GO:1903416
antibiotic	GO:0017000	GO:0046677, GO:0071236
methylglyoxal	GO:0019242	GO:0051595, GO:0097238
rhamnose	GO:0019300	GO:0032149
mannose	GO:0019307	GO:1905582, GO:1905583
hexose	GO:0019319	GO:0009746, GO:0140271
dethiobiotin	GO:0019351	GO:0044757, GO:1901690, GO:1905136
acetate	GO:0019413	GO:0010034, GO:0071311, GO:1901459
mannitol	GO:0019593	GO:0010555

l-ascorbic acid	GO:0019853, GO:2000082, GO:2000083	GO:0033591, GO:0071298
streptomycin	GO:0019872	GO:0046679, GO:0071239
peptide hormone	GO:0030072, GO:0090276, GO:0090277, GO:0090278	GO:0043434
insulin	GO:0030073, GO:0032024, GO:0046676, GO:0050796	GO:0032868
vasopressin	GO:0030103	GO:1904116, GO:1904117
bacteriocin	GO:0030152	GO:0046678, GO:0071237
growth hormone	GO:0030252, GO:0060123, GO:0060124, GO:0060125	GO:0060416
d-alanine	GO:0030632	GO:0170048
gonadotropin	GO:0032274, GO:0032276, GO:0032277, GO:0032278	GO:0034698
luteinizing hormone	GO:0032275, GO:0033684, GO:0033685, GO:0033686	GO:0034699, GO:0042700
hormone	GO:0032353, GO:0042446, GO:0046879, GO:0046883, GO:0046885, GO:0046886, GO:0046887, GO:0046888	GO:0009725
type i interferon	GO:0032479, GO:0032480, GO:0032481, GO:0032606, GO:0045351, GO:0072641	GO:0034340, GO:0071357
chemokine	GO:0032602, GO:0032642,	GO:1990868, GO:1990869

	GO:0032682, GO:0032722, GO:0042033, GO:0045073, GO:0045079, GO:0045080, GO:0050755, GO:0090195, GO:0090196, GO:0090197, GO:0090198	
granulocyte macrophage colony-stimulating factor	GO:0032604, GO:0032645, GO:0032685, GO:0032725, GO:0042253, GO:0045423, GO:0045424, GO:0045425	GO:0097012
hepatocyte growth factor	GO:0032605, GO:0032646, GO:0032686, GO:0032726, GO:0048175, GO:0048176, GO:0048177, GO:0048178	GO:0035728
interferon-alpha	GO:0032607, GO:0032647, GO:0032687, GO:0032727, GO:0045349, GO:0045354, GO:0045355, GO:0045356, GO:0072642, GO:1902739, GO:1902740, GO:1902741	GO:0035455, GO:0035457
interferon-beta	GO:0032608, GO:0032648, GO:0032688, GO:0032728, GO:0035546, GO:0035547, GO:0035548,	GO:0035456, GO:0035458

	GO:0035549, GO:0045350, GO:0045357, GO:0045358, GO:0045359	
type ii interferon	GO:0032609, GO:0032649, GO:0032689, GO:0032729, GO:0042095, GO:0045072, GO:0045077, GO:0045078, GO:0072643, GO:1902713, GO:1902714, GO:1902715	GO:0034341, GO:0060332, GO:0071346
interleukin-1	GO:0032612, GO:0032652, GO:0032692, GO:0032732, GO:0042222, GO:0045360, GO:0045361, GO:0045362, GO:0050701, GO:0050704, GO:0050711, GO:0050716	GO:0070555, GO:0071347
interleukin-11	GO:0032614, GO:0032654, GO:0032694, GO:0032734, GO:0042230, GO:0045363, GO:0045364, GO:0045365, GO:0072609, GO:0150169, GO:0150170, GO:0150171	GO:0071105, GO:0071348
interleukin-12	GO:0032615, GO:0032655, GO:0032695, GO:0032735, GO:0042090,	GO:0070671, GO:0071349

	GO:0045075, GO:0045083, GO:0045084, GO:0072610, GO:2001182, GO:2001183, GO:2001184	
interleukin-13	GO:0032616, GO:0032656, GO:0032696, GO:0032736, GO:0042231, GO:0045366, GO:0045367, GO:0045368, GO:0072611, GO:2000665, GO:2000666, GO:2000667	GO:0035962, GO:0035963
interleukin-15	GO:0032618, GO:0032658, GO:0032698, GO:0032738, GO:0042233, GO:0045372, GO:0045373, GO:0045374, GO:0072613	GO:0070672, GO:0071350
interleukin-17	GO:0032620, GO:0032660, GO:0032700, GO:0032740, GO:0042235, GO:0045378, GO:0045379, GO:0045380, GO:0072615, GO:1905076, GO:1905077, GO:1905078	GO:0097396, GO:0097398
interleukin-18	GO:0032621, GO:0032661, GO:0032701, GO:0032741, GO:0042241, GO:0045381,	GO:0070673, GO:0071351

	GO:0045382, GO:0045383, GO:0072616, GO:0150120, GO:0150121, GO:0150122	
interleukin-2	GO:0032623, GO:0032663, GO:0032703, GO:0032743, GO:0042094, GO:0045076, GO:0045085, GO:0045086, GO:0070970, GO:1900040, GO:1900041, GO:1900042	GO:0070669, GO:0071352
interleukin-21	GO:0032625, GO:0032665, GO:0032705, GO:0032745, GO:0042238, GO:0045390, GO:0045391, GO:0045392, GO:0072619	GO:0098756, GO:0098757
interleukin-3	GO:0032632, GO:0032672, GO:0032712, GO:0032752, GO:0042223, GO:0045399, GO:0045400, GO:0045401, GO:0072601	GO:0036015, GO:0036016
interleukin-4	GO:0032633, GO:0032673, GO:0032713, GO:0032753, GO:0042097, GO:0042224, GO:0045402, GO:0045403, GO:0045404, GO:0072602,	GO:0070670, GO:0071353

	GO:0150133,<br>GO:0150134,<br>GO:0150135	
interleukin-6	GO:0032635,<br>GO:0032675,<br>GO:0032715,<br>GO:0032755,<br>GO:0042226,<br>GO:0045408,<br>GO:0045409,<br>GO:0045410,<br>GO:0072604,<br>GO:1900165,<br>GO:2000778	GO:0070741,<br>GO:0071354
interleukin-7	GO:0032636,<br>GO:0032676,<br>GO:0032716,<br>GO:0032756,<br>GO:0042227,<br>GO:0045411,<br>GO:0045412,<br>GO:0045413,<br>GO:0072605,<br>GO:0150112,<br>GO:0150113,<br>GO:0150114	GO:0098760,<br>GO:0098761
interleukin-8	GO:0032637,<br>GO:0032677,<br>GO:0032717,<br>GO:0032757,<br>GO:0042228,<br>GO:0045414,<br>GO:0045415,<br>GO:0045416,<br>GO:0072606,<br>GO:2000482,<br>GO:2000483,<br>GO:2000484	GO:0098758,<br>GO:0098759
interleukin-9	GO:0032638,<br>GO:0032678,<br>GO:0032718,<br>GO:0032758,<br>GO:0042229,<br>GO:0045417,<br>GO:0045418,	GO:0071104,<br>GO:0071355

	GO:0045419, GO:0072607	
tumor necrosis factor	GO:0032640, GO:0032680, GO:0032720, GO:0032760, GO:0042533, GO:0042534, GO:0042535, GO:0042536, GO:1904467, GO:1904468, GO:1904469, GO:1990774	GO:0034612, GO:0071356
neurotrophin	GO:0032898, GO:0032899, GO:0032900, GO:0032901	GO:0038179
nerve growth factor	GO:0032902, GO:0032903, GO:0032904	GO:0038180, GO:0051394, GO:1990089
isoquinoline alkaloid	GO:0033075	GO:0014072, GO:0071317
raffinose	GO:0033529, GO:1900091, GO:1900092, GO:1900093	GO:0097403, GO:1901545
catecholamine	GO:0033604, GO:0033605, GO:0042423, GO:0050432, GO:0050433	GO:0071869
type iii interferon	GO:0034343, GO:0034344, GO:0034345, GO:0034346, GO:0072644	GO:0034342, GO:0071358
cortisol	GO:0034651, GO:0043400, GO:0051462, GO:0051463, GO:0051464, GO:2000064, GO:2000065, GO:2000066	GO:0051414

vitamin a	GO:0035238	GO:0033189, GO:0071299
parathyroid hormone	GO:0035898, GO:2000828, GO:2000829, GO:2000830	GO:0071107
steroid hormone	GO:0035929, GO:0090030, GO:0090031, GO:0090032, GO:0120178, GO:2000831, GO:2000832, GO:2000833	GO:0048545
corticosterone	GO:0035934, GO:2000852, GO:2000853, GO:2000854	GO:0051412
testosterone	GO:0035936, GO:0061370, GO:2000224, GO:2000225, GO:2000843, GO:2000844, GO:2000845	GO:0033574
estradiol	GO:0035938, GO:2000864, GO:2000865, GO:2000866	GO:0032355
dehydroepiandrosterone	GO:0035942, GO:2000840, GO:2000841, GO:2000842	GO:1903494, GO:1903495
melanocyte-stimulating hormone	GO:0036160	GO:1990680
macrophage colony-stimulating factor	GO:0036301, GO:1901256, GO:1901257, GO:1901258	GO:0036005, GO:0038145, GO:1902226, GO:1902227, GO:1902228, GO:1903971
sodium ion	GO:0036376, GO:0071436, GO:0098667, GO:1903273, GO:1903274, GO:1903275,	GO:0097369, GO:0098719, GO:1903782, GO:1903783, GO:1903784, GO:1990118

	GO:1903276, GO:1903277, GO:1903278	
bmp	GO:0038055, GO:1900144, GO:2001284, GO:2001285	GO:0008101, GO:0030509, GO:0030510, GO:0030513, GO:0030514, GO:0071772, GO:0090097, GO:0090098, GO:0090099
nicotine	GO:0042179	GO:0035094, GO:0071316
vitamin k	GO:0042371	GO:0032571, GO:0071307
phylloquinone	GO:0042372	GO:0032573, GO:0071309
long-chain fatty acid	GO:0042759	GO:0010746, GO:0010747, GO:0010748, GO:0015911
pheromone	GO:0042811	GO:0019236, GO:0071444
vitamin b6	GO:0042819	GO:0034516, GO:0071304
pyridoxal	GO:0042821	GO:0140204
l-alanine	GO:0042852	GO:1904273
mycotoxin	GO:0043386	GO:0010046, GO:0036146
corticotropin-releasing hormone	GO:0043396, GO:0043397, GO:0051465, GO:0051466	GO:0043435, GO:1900011
alkane	GO:0043447, GO:1901577, GO:1901578, GO:1901579	GO:1902778, GO:1902779, GO:1990373
puromycin	GO:0043638	GO:1905794, GO:1905795
tetracycline	GO:0043644	GO:0072746, GO:1901326
macrophage migration inhibitory factor	GO:0044807	GO:0035691, GO:2000446, GO:2000447, GO:2000448

adenine	GO:0046084, GO:0061934	GO:0061488, GO:0098702
guanine	GO:0046099	GO:0061489, GO:0098710
uracil	GO:0046107	GO:0098721, GO:1902431, GO:1905529, GO:1905530, GO:1905531
alcohol	GO:0046165, GO:1902930, GO:1902931, GO:1902932	GO:0097305, GO:0097306, GO:1901421, GO:1990335
methanol	GO:0046169	GO:0033986, GO:0071405
aldehyde	GO:0046184	GO:0110096
acetaldehyde	GO:0046186	GO:1905640, GO:1905641
cyanide	GO:0046202	GO:1903927, GO:1903928
toluene	GO:0046252	GO:1901424, GO:1901456
formaldehyde	GO:0046293	GO:1904404, GO:1904405
disaccharide	GO:0046351	GO:0034285
butyrate	GO:0043439, GO:0046358	GO:1903544, GO:1903545
monosaccharide	GO:0046364	GO:0034284
galactose	GO:0046369	GO:0140425
fructose	GO:0046370	GO:0009750, GO:0032445, GO:1990539
ceramide	GO:0046513, GO:1900060, GO:2000303, GO:2000304	GO:0106096, GO:0106097
tetrahydrofolate	GO:0046654	GO:1904481, GO:1904482
folic acid	GO:0046656	GO:0051593, GO:0071231
formic acid	GO:0046721	GO:1901425, GO:1901462
follicle-stimulating hormone	GO:0046880, GO:0046881,	GO:0032354, GO:0042699

	GO:0046882, GO:0046884	
arachidonate	GO:0050482, GO:0090237, GO:0090238, GO:1900139	GO:1904550, GO:1904551
cocaine	GO:0050799	GO:0042220, GO:0071314
pullulan	GO:0051677	GO:0044592, GO:1900520
l-proline	GO:0055129	GO:1903809, GO:1904271, GO:1905735, GO:1905736, GO:1905737
copper ion	GO:0060003	GO:0015678, GO:0046688, GO:0071280, GO:0098705, GO:1902861
tyramine	GO:0061545, GO:1901695	GO:0071928, GO:2000131, GO:2000132, GO:2000133
l-glutamine	GO:0062132, GO:0062133, GO:0062134, GO:1901704	GO:0036229, GO:1901034, GO:1901035, GO:1901036, GO:1903803, GO:1904844, GO:1904845
glucagon	GO:0070091, GO:0070092, GO:0070093, GO:0070094	GO:0033762
somatostatin	GO:0070253, GO:0090273, GO:0090274, GO:0090275	GO:0038170
lipoteichoic acid	GO:0070395	GO:0070391, GO:0071223
prolactin	GO:0070459, GO:1902721, GO:1902722	GO:0038161, GO:1902211, GO:1902212, GO:1902213,

		GO:1990637, GO:1990646
thyroid-stimulating hormone	GO:0070460, GO:2000612, GO:2000613, GO:2000614	GO:0038194
hydrogen sulfide	GO:0070814, GO:1904826, GO:1904827, GO:1904828	GO:1904880, GO:1904881
l-asparagine	GO:0070981	GO:0090469, GO:1903811
l-methionine	GO:0071265	GO:1903813, GO:1905544, GO:1905624, GO:1905625, GO:1905626
homocysteine	GO:0071268	GO:1905374, GO:1905375
transforming growth factor beta	GO:0038044, GO:0071604, GO:0071634, GO:0071635, GO:0071636, GO:2001201, GO:2001202, GO:2001203	GO:0071559
chemokine (c-c motif) ligand 5	GO:0071609, GO:0071649, GO:0071650, GO:0071651	GO:0035689
granulocyte colony-stimulating factor	GO:0071611, GO:0071655, GO:0071656, GO:0071657	GO:0038158, GO:1990638, GO:1990643
purine-containing compound	GO:0072522	GO:0014074, GO:0071415
interleukin-32	GO:0072637, GO:0072638, GO:0150188, GO:0150189, GO:0150190, GO:0150191, GO:0150192, GO:0150193, GO:0150194	GO:0097395, GO:0097397

fibroblast growth factor	GO:0090269, GO:0090270, GO:0090271, GO:0090272	GO:0071774
platelet-derived growth factor	GO:0090360, GO:0090361, GO:0090362	GO:0036119
l-glutamate	GO:0097054	GO:0002036, GO:0002037, GO:0002038, GO:0098712, GO:1900920, GO:1900921, GO:1900922, GO:1902065, GO:1903802, GO:1905232, GO:1990123
leukotriene b4	GO:0097251	GO:1905389, GO:1905390
morphine	GO:0097295	GO:0043278, GO:0071315
chemokine (c-c motif) ligand 19	GO:0097388	GO:0038115
chemokine (c-c motif) ligand 21	GO:0097389	GO:0038116
chemokine (c-x-c motif) ligand 12	GO:0097390	GO:0038146
potassium ion	GO:0071435, GO:0097623, GO:0098668, GO:1902302, GO:1902303, GO:1902304, GO:1903764, GO:1903765, GO:1903766	GO:0010107, GO:0035864, GO:0035865, GO:1903287, GO:1903288, GO:1990573
histidine	GO:0120213, GO:0120214, GO:0120215	GO:0071232, GO:0080052
manganese ion	GO:0140048	GO:0010042, GO:0071287
fluoride	GO:0140116	GO:1902617, GO:1902618
mycophenolic acid	GO:0140722	GO:0071505, GO:0071506
benzene	GO:1900997	GO:1901423, GO:1901453

erythromycin	GO:1901115	GO:0072743, GO:1901323
ether	GO:1901503	GO:0045472, GO:0071362
strigolactone	GO:1901601	GO:1902347, GO:1902348
calcium ion	GO:1901660, GO:1905912, GO:1905913, GO:1905914, GO:1990034	GO:0051592, GO:0071277, GO:0098703, GO:1905664, GO:1905665, GO:1905947, GO:1905949, GO:1990035
cannabinoid	GO:1901696	GO:0038171
l-isoleucine	GO:1901705	GO:0090476, GO:0090477, GO:1903806
quercetin	GO:1901734	GO:1905235, GO:1905236
beta-carotene	GO:1901812	GO:1905387, GO:1905388
astaxanthin	GO:1901815	GO:1905217, GO:1905218
isobutanol	GO:1901961	GO:1902665, GO:1990337
l-dopa	GO:1903185, GO:1903195, GO:1903196, GO:1903197	GO:1904473, GO:1904474
glyoxal	GO:1903191	GO:0036471
reactive oxygen species	GO:1903409, GO:1903426, GO:1903427, GO:1903428	GO:0000302, GO:0034614, GO:1901033
iron ion	GO:1903414, GO:1903988	GO:0010039, GO:0071281, GO:0097459, GO:0097460, GO:0098707, GO:0098711, GO:1903989, GO:1903990, GO:1903991, GO:1904438,

		GO:1904439, GO:1904440
endothelin	GO:1904470, GO:1904471, GO:1904472, GO:1990775	GO:1990839, GO:1990859

Appendix D. Supplementary figures showing representative interaction terms with approximately zero or negative causal enrichment scores (CES) across four datasets.

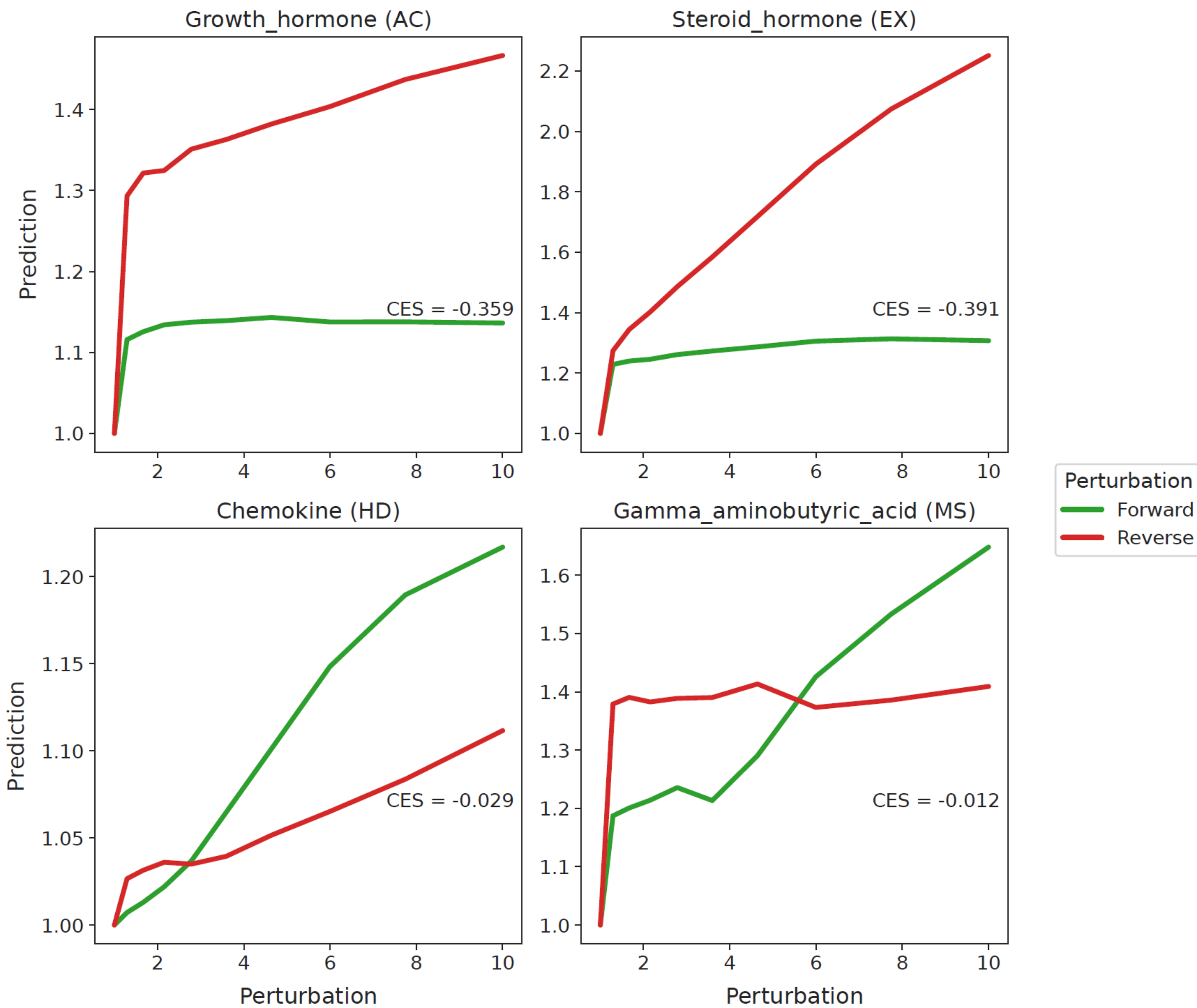

Supplement: Supplementary file 1 [file media-1.pdf]
